# Effects of Time and Duration of Progesterone Administration on Mammary Tumours Induced by 7,12-Dimethylbenz(a)Anthracene in Sprague-Dawley Rats

**DOI:** 10.1038/bjc.1973.8

**Published:** 1973-01

**Authors:** Anne G. Jabara, P. H. Toyne, Alison G. Harcourt

## Abstract

A reduction in tumour yield was apparent when progesterone administration was begun 25 days before feeding 7,12-dimethylbenz(a)anthracene (DMBA). This effect was most obvious when the duration of hormone administration was brief. Continuation of progesterone for some time after feeding DMBA caused a progressive diminution of the inhibitory effect, and 135 days of continuous hormone treatment entirely abolished the effects of 25 days pretreatment with the hormone.

In contrast, when progesterone injections were begun 2 days after feeding DMBA, there was a trend towards enhancement of tumour yield. Continuous hormone administration appeared more effective than shorter treatment regimens.


Br. J. Cancer (1973) 27, 63

### EFFECTS OF TIME AND DURATION OF PROGESTERONE ADMINISTRATION ON MAMMARY TUMOURS INDUCED BY 7,12-DIMETHYLBENZ(a)ANTHRACENE IN SPRAGUE–DAWLEY RATS

ANNE G. JABARA, P. H. TOYNE AND ALISON G. HARCOURT

*From the Departments of Pathology and Statistics, University of Melbourne, Parkville, Victoria* 3052

Received 7 August 1972. Accepted 1 September 1972

**Summary.—A reduction in tumour yield was apparent when progesterone administration was begun 25 days before feeding 7,12-dimethylbenz(a)anthracene (DMBA). This effect was most obvious when the duration of hormone administration was brief. Continuation of progesterone for some time after feeding DMBA caused a progressive diminution of the inhibitory effect, and 135 days of continuous hormone treatment entirely abolished the effects of 25 days pretreatment with the hormone.**

**In contrast, when progesterone injections were begun 2 days after feeding DMBA, there was a trend towards enhancement of tumour yield. Continuous hormone administration appeared more effective than shorter treatment regimens.**

PREVIOUS investigations have shown that exogenous progesterone, while not carcinogenic *per se*, significantly enhances 7,12-dimethylbenz(a)anthracene (DMBA) mammary tumorigenesis in entire rats. This effect was shown when continuous hormone injections were begun 2 days before or 15 days after carcinogen administration or after the first tumour had appeared (Jabara, 1967; Jabara and Harcourt, 1970), or when daily injections of progesterone were given for only 30 days, beginning 15 days after feeding DMBA (Huggins, Moon and Morii, 1962). In contrast, Welsch, Clemens and Meites (1968) reported that prolonged (25 days) daily treatment with progesterone before administering DMBA significantly inhibited mammary tumorigenesis, even although hormone injections were continued for 15 days after carcinogen administration.

The present experiments were designed to determine the effect of variations in the time at which hormone administration was begun, and in the duration of hormone treatment, on the enhancing effect of progesterone on DMBA mammary tumorigenesis.

### MATERIALS AND METHODS

One hundred and forty-eight non-inbred Sprague–Dawley virgin female rats were divided randomly into 8 progesterone-treated groups of 16 rats each and one control group of 20 animals (Table I). They were housed 5 rats/cage and fed commercial pellets and water *ad libitum*. At 50 days of age each rat in all 9 groups was fed by gastric intubation with a single dose of 30 mg of DMBA (Eastman Organic Chemicals, U.S.A.) dissolved in 2 ml of corn oil. In addition, each animal in Groups 2–9 received subcutaneous injections of 3 mg of progesterone (Sigma Chemical Co., U.S.A.) dissolved in 0·1 ml of corn oil/day 3 times a week. In Groups 2–5, progesterone injections were begun 25 days before DMBA administration (*i.e.* on their 25th day of age) (DMBA + P − 25) and were continued for 18, 36, 54 and 160 days for Groups 2–5 respectively. In Groups 6–9, hormone injections were begun 2 days after feeding DMBA

(*i.e.* on their 52nd day of age) (DMBA + P + 2) and were continued for 9, 18, 27 and 133 days for Groups 6–9 respectively. Allocation of the 9 groups to the possible factor combinations is summarized as follows:

| Time of beginning progesterone treatment | | No treatment with progesterone | | Duration of progesterone treatment | |
|---|---|---|---|---|---|
| | | | | Short | Continuous |
| DMBA only | . | 1 | . | — | — |
| DMBA+P−25 | . | — | . | 2, 3, 4 | 5 |
| DMBA+P+2 | . | — | . | 6, 7, 8 | 9 |

Beginning 4 weeks after DMBA administration, all rats were palpated weekly and any mammary tumours recorded, measured and graphed as described previously (Jabara, 1967). Portions of each mammary tumour were removed at autopsy, fixed in 10% buffered formalin and 5 $\mu$m paraffin sections were stained with haematoxylin and eosin.

The statistical analysis of the results was based on the following 7 parameters of tumour yield: tumour incidence (the number of rats developing a tumour, of those which survived longer than 28 days after DMBA administration), latent period (the time interval between feeding DMBA and the appearance of the first tumour), average number of active tumour centres per rat (the number of tumours per rat which were palpable for at least 5 weeks and/or were visible macroscopically at autopsy averaged over all rats in a group, including those rats which did not develop a neoplasm), tumour growth behaviour and size, tumour locations and types of neoplasm developed (Jabara, 1967).

The first 2 of these parameters, tumour incidence and latent period, together with the date of death of rats which died from other causes before any tumour had developed, determined the shape of the actuarial survival curve in which the proportion of rats still alive and without any tumours was plotted against the time lapsed since administration of DMBA (Pike and Roe, 1963). By using the curve derived from the results for the 147 rats which survived more than 28 days after feeding DMBA, the number of rats in each group expected to develop a tumour under the null hypothesis of no difference between groups can be estimated (Roe *et al.*, 1970).

The remaining 5 parameters were not suitable for analysis by the actuarial method, and standard statistical techniques were therefore used in their analysis. The average numbers of active tumour centres developed per rat were compared by an analysis of variance, the data first being transformed using a square root transformation. An arcsin transformation was used for comparisons of tumour size between the several groups. In all other cases, the $\chi^2$-test was used for comparisons between groups.

The overall effect of the starting time for progesterone treatment was assessed by comparing Groups 2–5 and Groups 6–9 with each other and with Group 1. The effect of the duration of progesterone treatment was examined within the DMBA + P − 25 groups by comparing Groups 2, 3, 4 and 5, and within the DMBA + P + 2 groups by comparing Groups 6, 7, 8 and 9. The possibility of interaction between starting time and duration of hormone treatment was examined by comparing each of Groups 2–4, Group 5, Groups 6–8 and Group 9 with the control Group 1.

### RESULTS

No mammary tumour was observed to develop during the first 4 weeks following carcinogen administration. Twenty-three rats in the 8 progesterone-treated groups died or were killed between the 4th week and the end of the experiment (135 days after feeding DMBA) (Table I). The average survival times for these rats were 110, 120, 94, 96·5, 76, 99, 75 and 88 days in Groups 2–9 respectively. Twelve of these rats had developed tumours before death, and 11 were tumour free. All rats in the control group which were alive 28 days after receiving DMBA survived for the remainder of the experiment.

### *Tumour incidence*

The observed tumour incidence in each group was compared with the corresponding expected incidence under the

TABLE II.—*Observed and (Actuarially) Expected Incidence of Tumours*

| Time of beginning progesterone treatment | | No treatment with progesterone | Duration of progesterone treatment | | | |
|---|---|---|---|---|---|---|
| | | | Short | | | Continuous |
| DMBA only | O | 13 | — | — | — | — |
| | E | 12·76 | — | — | — | — |
| | R | 1·04 | — | — | — | — |
| DMBA+$P$−25 | O | — | 6 | 7 | 8 | 11 |
| | E | — | 12·49 | 12·67 | 10·30 | 9·26 |
| | R | — | 0·49 | 0·56 | 0·79 | 1·21 |
| DMBA+$P$+2 | O | — | 11 | 11 | 9 | 13 |
| | E | — | 6·39 | 9·80 | 9·50 | 5·82 |
| | R | — | 1·76 | 1·14 | 0·97 | 2·28 |

O = observed incidence; E = expected incidence; R = relative incidence rate (O/E).

null hypothesis of no difference between groups. The expected incidences were calculated using the actuarial survival curve following the method described by Roe *et al.* (1970). The observed (O) and expected (E) incidences, and the relative incidence rates (R = O/E) are shown in Table II. The $\chi^2$ value for Table II was 19·1, with 8 degrees of freedom ($P < 0{\cdot}02$). Using the actuarial method, the estimated probability that a rat would develop at least one tumour during the experimental period has been calculated for each group (Table I).

The relative incidence rates indicated that pretreatment with progesterone inhibited tumorigenesis, except in the group (5) in which progesterone treatment was continued for the duration of the experiment (Table II). Beginning hormone administration 2 days after feeding DMBA enhanced tumorigenesis in 3 of the 4 groups so treated (Group 8 being the exception); the effect was again particularly marked in the group (9) in which progesterone was given throughout the whole experiment (Table II).

Of the 6 groups of rats which received progesterone for only short periods, there appeared to be a progressive increase in tumorigenesis with increasing duration of hormone treatment in the 3 hormone pretreated groups (2, 3 and 4), but tumorigenesis did not show a progressive increase with increasing length of hormone treatment in Groups 6, 7 and 8, in which progesterone injections were begun 2 days after DMBA administration (Table II).

### *Active tumour centres*

The analysis of the results observed for this parameter was based on the figures recorded for the 124 rats which survived for the whole experimental period. The average number of active tumour centres per rat (ATCs) was based on all rats in a group, including those which did not develop a neoplasm.

A significantly higher average number of ATCs arose in Groups 6–9 compared with the average number in Groups 2–5 (Table I and IV). However, when the average number of ATCs in Groups 6–9 and in Groups 2–5 were each compared separately with the controls, the differences were not significant (Table I and IV).

The average numbers of ATCs obtained in Groups 2, 3 and 4 did not differ significantly from each other, nor was the overall average for these 3 groups significantly lower than that observed in Group 5. However, the pooled average for Groups 2–4 was significantly lower than the pooled average for Groups 6–8 and that observed in the controls (Table I and IV). There was no significant difference between the average numbers of ATCs per rat observed in Groups 6, 7 and 8, nor did the pooled average for

TABLE III.—*Tumour Growth Behaviour, Locations and Histological Tumour Types Induced*

| | Group no. | | | | | | | | |
|---|---|---|---|---|---|---|---|---|---|
| | 1 | 2 | 3 | 4 | 5 | 6 | 7 | 8 | 9 |
| Growth behaviour of tumours: | | | | | | | | | |
| No. classified CG | 3 | 1 | 5 | 1 | 8 | 7 | 3 | 5 | 5 |
| No. classified S | 9 | 5 | 7 | 10 | 13 | 5 | 7 | 6 | 23 |
| No. classified R | 8 | 0 | 1 | 2 | 6 | 2 | 1 | 1 | 7 |
| No. unclassified* | 12 | 4 | 6 | 3 | 5 | 11 | 10 | 3 | 14 |
| No. of measurable tumours | 3 | 3 | 6 | 3 | 17 | 9 | 5 | 6 | 14 |
| No. of rats bearing measurable tumours | 3 | 3 | 5 | 3 | 10 | 7 | 3 | 5 | 6 |
| Locations of tumours: | | | | | | | | | |
| No. in anterior 3 pairs | 22 | 9 | 15 | 12 | 26 | 20 | 18 | 12 | 30 |
| No. in posterior 3 pairs | 10 | 1 | 4 | 4 | 6 | 5 | 3 | 3 | 19 |
| Histological tumour types: | | | | | | | | | |
| No. classified carcinoma | 28 | 7 | 15 | 12 | 22 | 22 | 17 | 9 | 33 |
| No. classified (fibro)adenoma | 3 | 1 | 0 | 0 | 5 | 1 | 1 | 1 | 6 |
| No. unclassified† | 1 | 2 | 4 | 4 | 5 | 2 | 3 | 5 | 10 |

* Growth behaviour could not be classified as either no measurements or insufficient measurements of tumours were obtained before death of the host.
† Tumours could not be classified due to complete regression before autopsy.
CG = continuous growth
S = static growth
R = regressing

TABLE IV.—*Summary of Statistical Analyses*

| Group comparisons | Active tumour centres | Tumour growth behaviour | Tumour size | Tumour locations |
|---|---|---|---|---|
| 1 v 2–5 | NS | NS | $P < 0{\cdot}001$ | NS |
| 1 v 6–9 | NS | $P = 0{\cdot}048$ | $P = 0{\cdot}003$ | NS |
| 2–5 v 6–9 | $P = 0{\cdot}003$ | NS | NS | NS |
| 2–4 v 6–8 | $P = 0{\cdot}027$ | NS | NS | NS |
| 5 v 9 | $P = 0{\cdot}022$ | NS | $P = 0{\cdot}026$ | NS |
| 2–4 v 5 | NS | NS | $P = 0{\cdot}006$ | NS |
| 6–8 v 9 | $P = 0{\cdot}005$ | $P = 0{\cdot}041$ | NS | $P = 0{\cdot}027$ |
| 1 v 2–4 | $P = 0{\cdot}026$ | $P = 0{\cdot}031$ | $P = 0{\cdot}033$ | NS |
| 1 v 5 | NS | NS | $P < 0{\cdot}001$ | NS |
| 1 v 6–8 | NS | $P = 0{\cdot}019$ | $P = 0{\cdot}003$ | NS |
| 1 v 9 | $P = 0{\cdot}011$ | NS | $P = 0{\cdot}013$ | NS |

For histological tumour types developed all of the above comparisons were found to be not significant (NS).

these 3 groups differ significantly from that for the controls. Likewise, the average number of ATCs per rat in Group 5 did not differ significantly from that observed in the controls. However, all 3 groups, *i.e.* the controls, Group 5 and the pooled group containing the original Groups 6–8, gave values which were significantly lower than the average number of ATCs per rat in Group 9 (Table IV).

*Tumour growth behaviour and tumour size*

The results were based on all observed tumours, including those which developed in rats which died or were killed before the end of the experiment. With the exception of Group 2, in which no regressing tumours were observed, 3 main types of tumour growth behaviour (TGB) (Jabara, 1967) occurred in animals of all groups (Table III).

The TGB of classifiable neoplasms in all progesterone-treated groups (2–9) was significantly different from that in the controls ($P = 0{\cdot}027$), and was due to a relatively high number of regressing tumours in Group 1 (Table III).

Investigations into the effect of the duration of progesterone administration on TGB showed that the TGB in Groups

6–8, receiving progesterone for relatively short periods, was significantly different from that in Group 9 (Table III and IV). This difference was ascribed to a relative excess of continuously growing neoplasms and a relative shortage of regressing tumours in Groups 6–8 compared with those in Group 9 (Table III). Groups 6–8 and Groups 6–9 also differed significantly from the controls with respect to this parameter (Table III and IV), again due to a relative excess of continuously growing tumours in the first 3 groups and to a relative excess of regressing neoplasms in the controls (Group 1). While the TGB of Groups 2–4 was not significantly different from that in Group 5, it differed significantly from that in the controls (Table III and IV). This difference was based on relatively small numbers of classifiable neoplasms (32 in Groups 2–4 and 20 in Group 1), but again it appeared to be due to a relative excess of regressing growths in Group 1.

No other significant effects on TGB were found (Table IV).

A highly significant difference was observed between the number of tumours which reached a measurable size (1cm or more in longest diameter) of all those that developed in the control group (1) compared with those arising in the 8 progesterone-treated groups ($P < 0{\cdot}01$) (Table III and IV). This difference was ascribed to the small proportion of measurable tumours developed in Group 1. Within the 8 groups treated with various progesterone regimens, Group 5 was the only one which showed a significantly higher incidence of measurable tumours compared with that in Groups 2–4 and 6–8 combined ($P = 0{\cdot}010$) and that in Group 9; it was also significantly higher than that in Group 1 (Table IV).

*Location*

In all 9 groups, neoplasms arose equally on both sides. The majority of growths in all groups developed in the anterior 3 pairs of glands, and less often in the 4th, 5th and 6th pairs (Table III). However, comparisons between the numbers of tumours arising in the first 3 pairs of mammae and the numbers developing in the last 3 pairs revealed that while Group 9 was not significantly different from the controls, this group differed significantly not only from groups 6–8 (Table IV), but from all the other progesterone-treated groups ($P = 0{\cdot}020$). This difference was due to a relative excess of neoplasms arising in the 3 pairs of abdominal glands in Group 9 (Table III).

*Types of tumours*

The classifiable tumours arising in rats of all groups consisted of both benign and malignant growths (Jabara, 1967), except in Groups 3 and 4 where only carcinomata arose (Table III). Progesterone appeared not to influence either the macroscopic or microscopic appearances of the developing tumours, regardless of when injections were begun or for how long they were continued. Comparisons of the proportions of benign to malignant tumours developed per group showed that the 2 groups receiving progesterone continuously (5 and 9) developed significantly more benign tumours than the 6 groups treated with the hormone for only short periods ($P = 0{\cdot}031$) (Table III). However, these 2 groups did not differ significantly from each other, or from Group 1, in this regard.

### DISCUSSION

Results obtained in the DMBA+P—25 groups in the present series confirmed the findings of Welsch *et al.* (1968) that prolonged pretreatment with progesterone before feeding DMBA markedly reduced the incidence of mammary tumours if progesterone administration is continued only for a short time after carcinogen administration. However, the data have also shown that an even greater inhibition of mammary tumorigenesis was obtained by stopping hormone administration before feeding DMBA, and further that the longer progesterone was administered after feeding DMBA, the progressively less obvious became the inhibitory effect of hormone pretreatment on tumorigenesis; 135 days of continuous progesterone administration after DMBA abolished the inhibitory effect of 25 days of hormone pretreatment.

Hormone pretreatment, as shown in the series of Welsch and his colleagues, resulted in a significant reduction in the number of active tumour centres per rat in the 3 groups (2–4) receiving only short post-treatment with the hormone compared with those in the controls. However, the inhibitory effect of 25 days of hormone pretreatment was abolished by continuing progesterone administration for 135 days after feeding DMBA. Tumours were not weighed in the present series, but the fact that tumour size, compared with that in the controls, was significantly increased in all progesterone-treated groups, regardless of the time of its administration or its duration, strongly suggests that the total weight of tumours per rat was probably also increased, contrasting with the significant reduction in tumour weight observed by Welsch *et al.* (1968).

With one exception, both short and continuous post-treatment with progesterone (DMBA + P + 2) was found to enhance the mammary tumour incidence compared with the controls, continuous administration being much more effective in this regard than shorter regimens. Group 8 was exceptional in that its tumour incidence approximated that observed in the controls. The reason for lack of tumour enhancement in this group is inexplicable. In contrast to the pretreated groups, tumorigenesis did not show a progressive increase with increasing duration of hormone treatment in the short post-treated groups (6–8). One explanation for this observation could be due to the shorter (9-day) time intervals between stopping the different hormone regimens in Groups 6–8, compared with 18-day intervals in the pretreated Groups 2–4, the longer time interval in the latter groups probably allowing differences between treatment regimens to become more obvious.

The previously reported significant enhancement of active tumour centres per rat following continuous post-treatment with progesterone (Jabara, 1967; Jabara and Harcourt, 1970) was confirmed in Group 9 of the present series. However, unlike Huggins *et al.* (1962), who reported a significant enhancement of ATCs per rat following short (30 days) hormone post-DMBA treatment, only a trend towards enhancement of ATCs was observed in Group 6 of the present series, whereas in the other 2 groups also receiving short post-treatment with progesterone there was a non-significant trend towards a reduction in ATCs developed per rat. The explanation for the differences between the present series and that of Huggins and colleagues may lie in the length of the experimental period. The present experiment was terminated 135 days after feeding DMBA compared with 180 days in that of Huggins *et al.* (1962). In rats fed only DMBA, the majority of benign tumours tends to arise several weeks or months later than do malignant ones (Daniel and Prichard, 1964; Jabara, 1967). In contrast, administration of progesterone in addition to DMBA tends to hasten the appearance of benign, as well as malignant, growths (Jabara, 1967). Hence, the longer the experimental period, the more apparent become the differences in the various parameters of tumour yield between groups of animals treated with DMBA alone and those treated with progesterone as well. The relatively short experimental period used in the present series probably also explains the non-significant increase in benign tumours observed in the 2 groups (5 and 9) which received progesterone continuously compared with the number arising in the controls. However, the experimental period was long enough to demonstrate that continuous hormone administration resulted in the appearance of significantly more benign growths than did shorter hormone regimens. In view of the fact that continuous progesterone administration in previous series (Jabara, 1967; Jabara and Harcourt, 1970) and in Group 5 (DMBA + *P* — 25 to + 135) of the present experiments has not been found to influence significantly the locations of neoplasms, it is suggested that the significant increase in the number of tumours arising in the abdominal pairs of mammary glands in Group 9 (DMBA + P + 2 to + 135) probably can be ascribed to a random effect.

Continuous post-treatment with progesterone in previous investigations did not result in any demonstrable modification of tumour growth behaviour (Jabara, 1967; Jabara and Harcourt, 1970), although Huggins *et al.* (1962) reported an increase in tumour growth rate in rats injected with progesterone from +15 to +45 days after carcinogen administration. The findings in the present series confirm those of Huggins and his colleagues and further show that the presence of progesterone, regardless of the time of beginning its administration or its duration, appears to promote tumour growth as assessed by both tumour growth behaviour and tumour size. The significantly higher incidence of measurable tumours in Group 5 (DMBA + *P* — 25 to + 135) compared with those in the other 7 progesterone regimens, probably reflects the long period (160 days) of hormone treatment in this group compared with 133 days in Group 9 and between 9 and 54 days duration in the other 6 hormone-treated groups.

The observation that neither short nor continuous post-treatment with progesterone (Groups 6–9) significantly modified the types of tumours developed confirms the findings in previous investigations (Huggins *et al.*, 1962; Jabara and Harcourt, 1970).

It is concluded that when progesterone administration was begun 2 days after feeding DMBA, regardless of its duration, it resulted in a trend towards an enhancement of several parameters of tumour yield, TGB and tumour size both being significantly enhanced. Continuous post-treatment with the hormone appeared much more effective than shorter treatment regimens. However, pretreatment with progesterone, particularly when begun early and administered for only a short period, decreased DMBA tumorigenesis. The mechanism whereby long progesterone pretreatment produces this effect is not yet known. Prolonged treatment with progesterone causes marked lobular–alveolar development of the rat mammary gland and Welsch *et al.* (1968) suggested that this enhanced mammary development close to the time of administering DMBA rendered the gland relatively refractory to carcinogen action. However, investigations have shown both the level of DNA synthesis in mammary epithelial cells and the extent of mammary gland development close to the time of carcinogen administration to be similar in 2 groups of rats given progesterone, beginning at either 25 or 2 days before feeding DMBA (Jabara, Toyne and Fisher, 1972); continuous progesterone administration when begun 2 days before feeding the carcinogen significantly enhances DMBA tumorigenesis (Jabara, 1967; Jabara and Harcourt, 1970). These observations therefore fail to confirm the suggestion advanced by Welsch and his colleagues. Dao (1971), on the other hand, has suggested that the presence of excessive amounts of steroid hormone at receptor sites in mammary epithelial cells may block interaction between DMBA and these sites and hence inhibit the induction of mammary tumours. In view of the fact that the rat rapidly metabolizes progesterone (Davies and Ryan, 1972), this hypothesis fails to explain why the greatest depression in overall tumour yield occurred in the pretreated group (2) in which progesterone administration was stopped 7 days before feeding DMBA. It appears that long progesterone pretreatment combined with DMBA administration could serve as a useful model for tumour initiation studies.

The authors wish to thank Dr J. S. Maritz, Department of Statistics, University of Melbourne, for helpful discussions concerning the statistical analysis. This work was carried out during the tenure of a grant from the Anti-Cancer Council of Victoria to one of us (A. G. J.).

### REFERENCES

Daniel, P. M. & Prichard, M. M. L. (1964) Three Types of Mammary Tumour Induced in Rats by Feeding with DMBA. *Br. J. Cancer*, **18**, 513.

Dao, T. L. (1971) Inhibition of Tumor Induction in Chemical Carcinogenesis in the Mammary Gland. *Progr. exp. Tumor Res.*, **14**, 59.

Davies, I. J. & Ryan, K. J. (1972) The Uptake of Progesterone by the Uterus of the Pregnant Rat *in vivo* and Its Relationship to Cytoplasmic Progesterone-binding Protein. *Endocrinology*, **90**, 507.


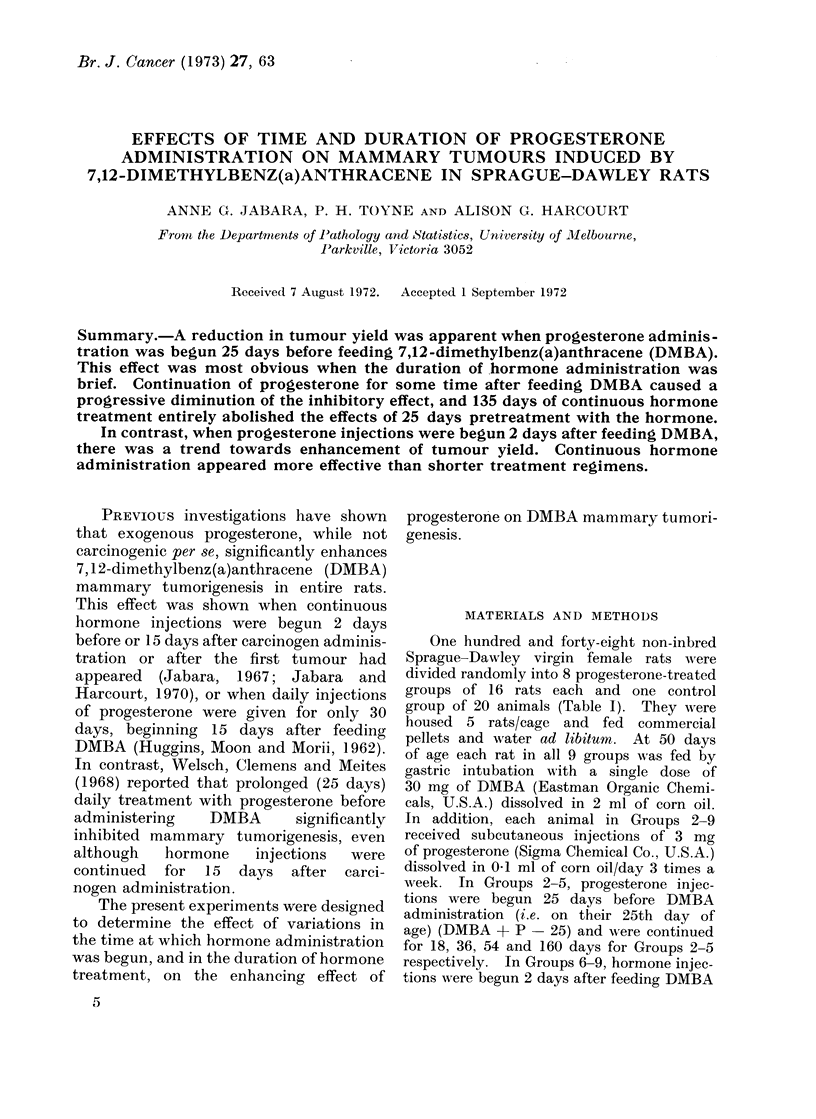

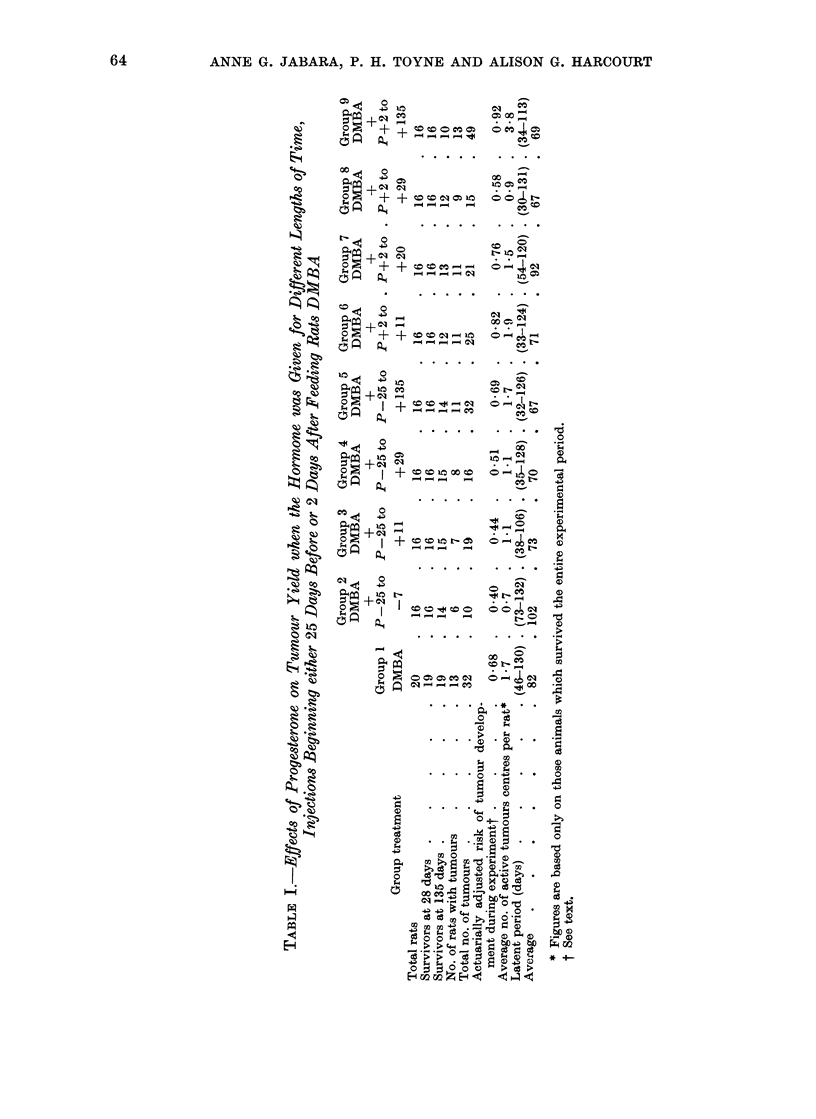

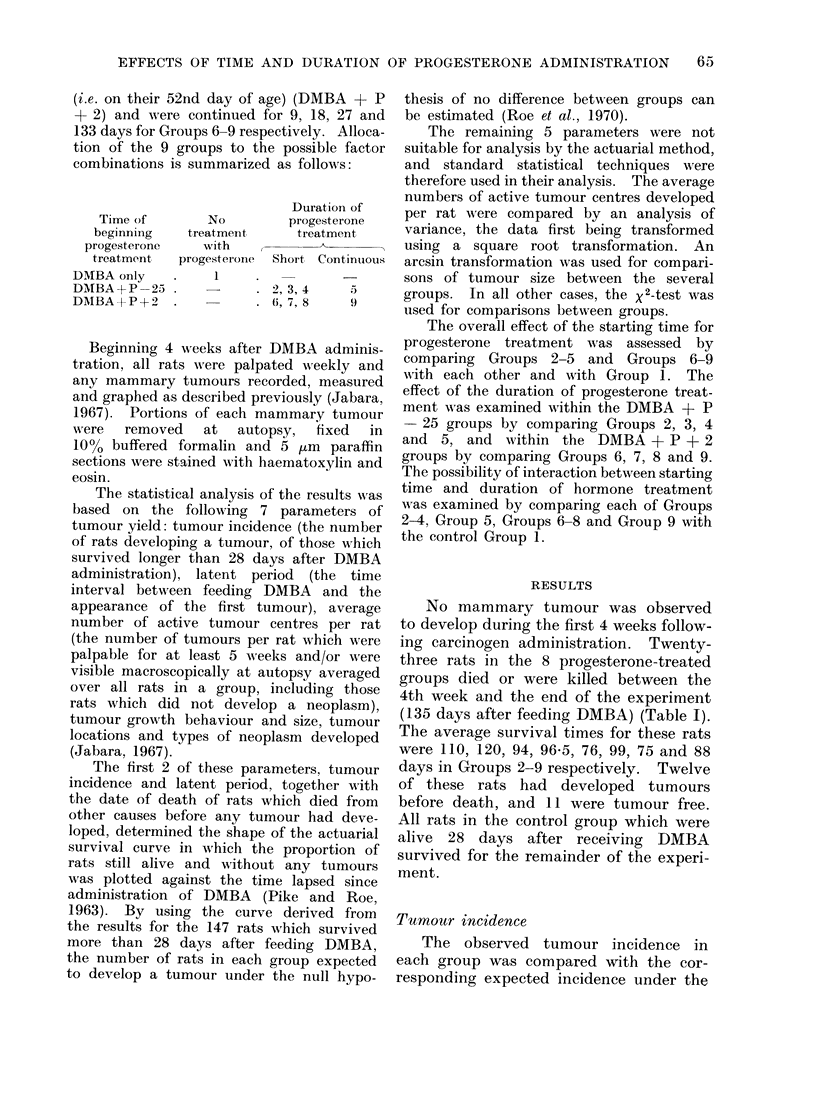

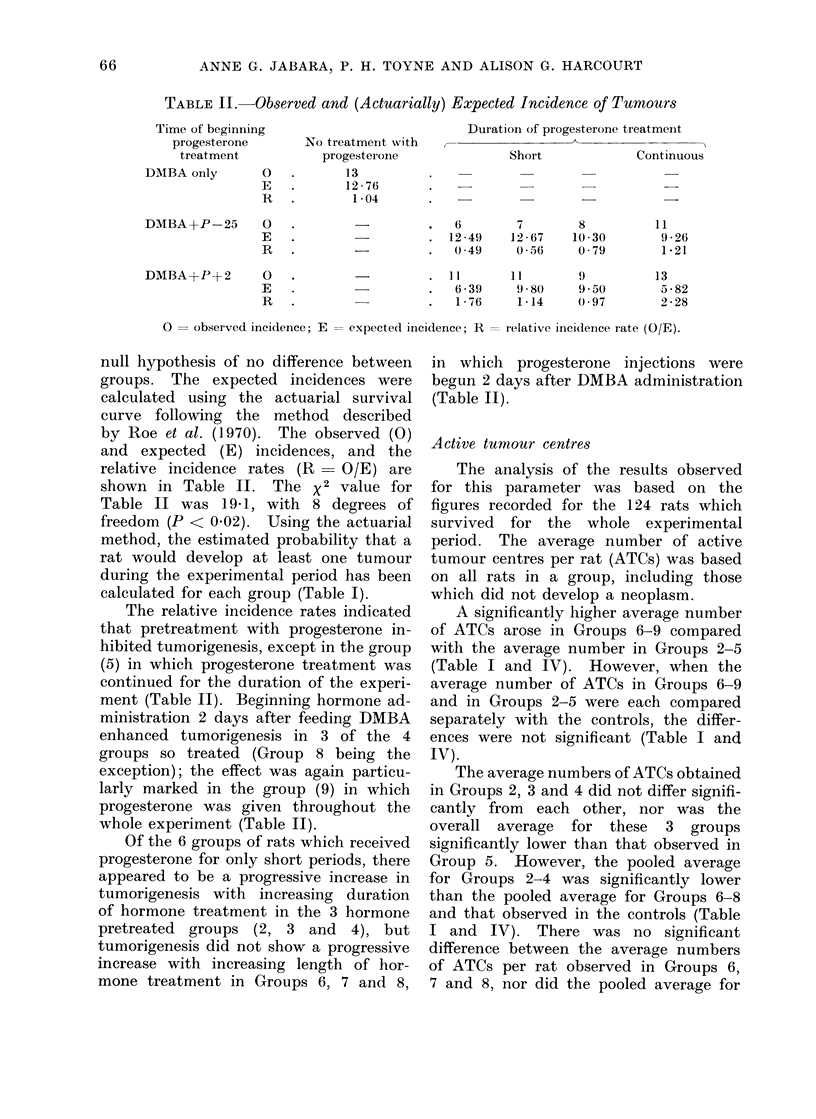

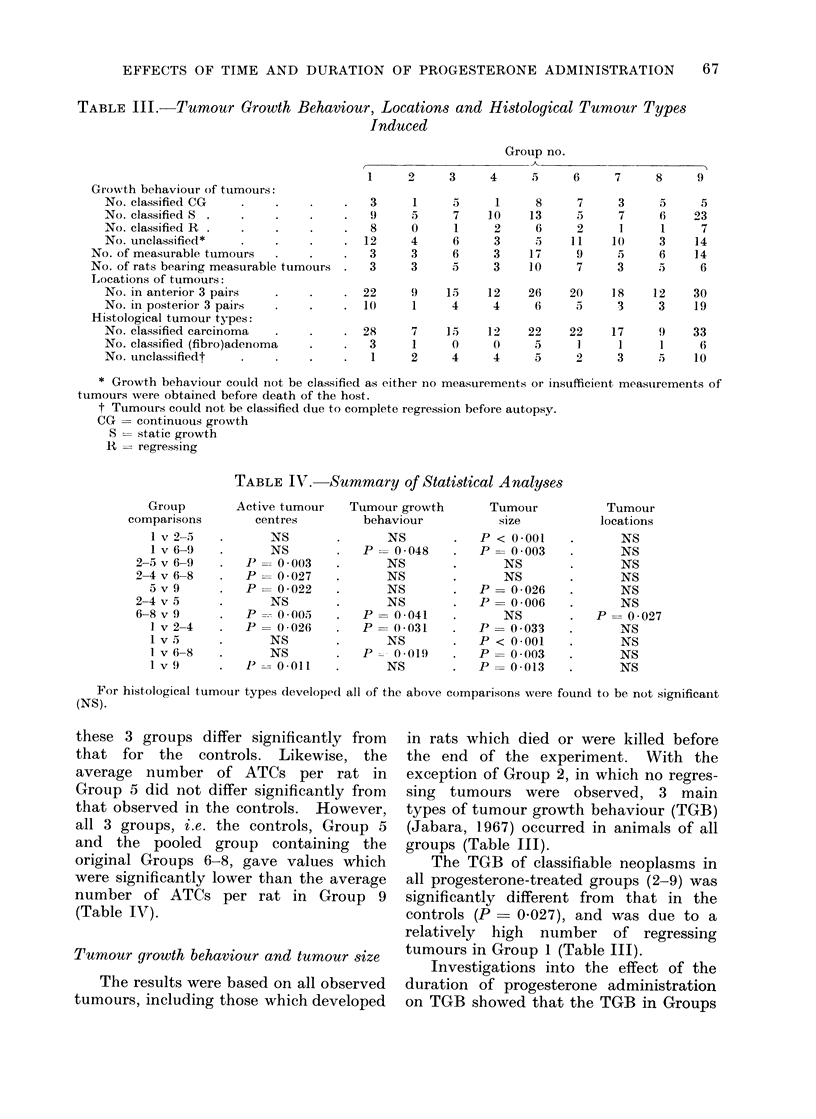

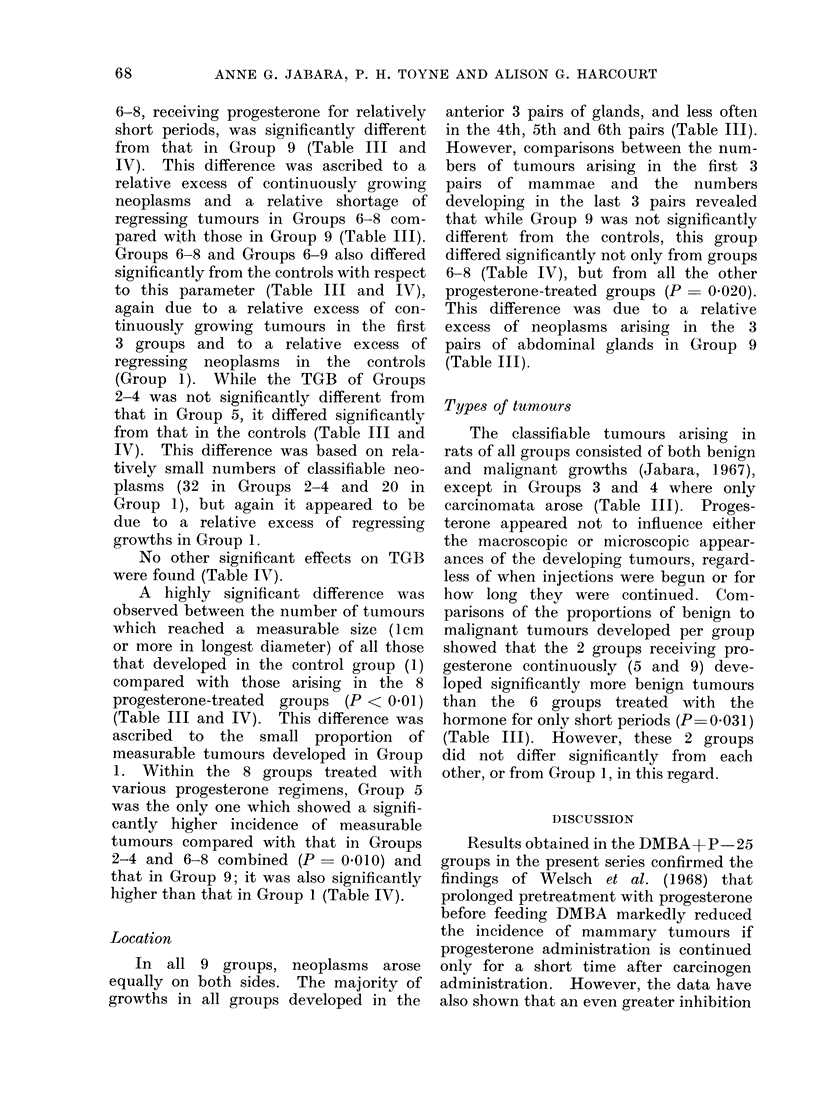

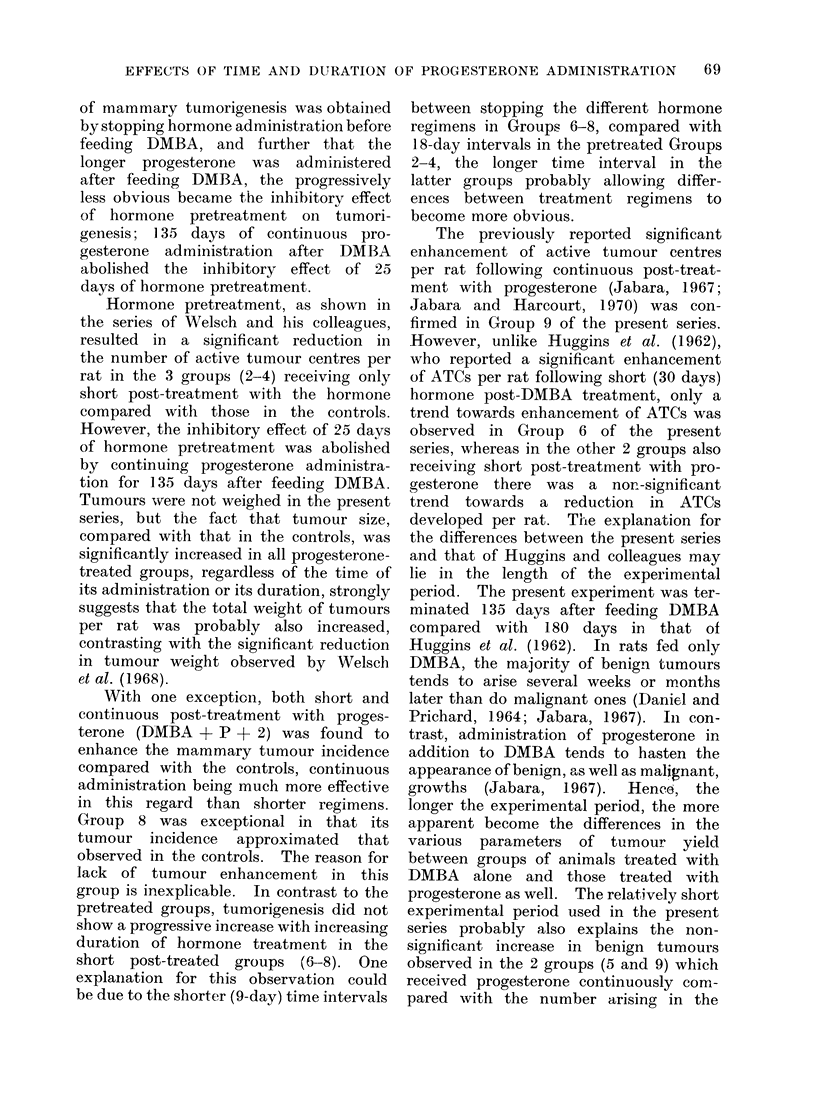

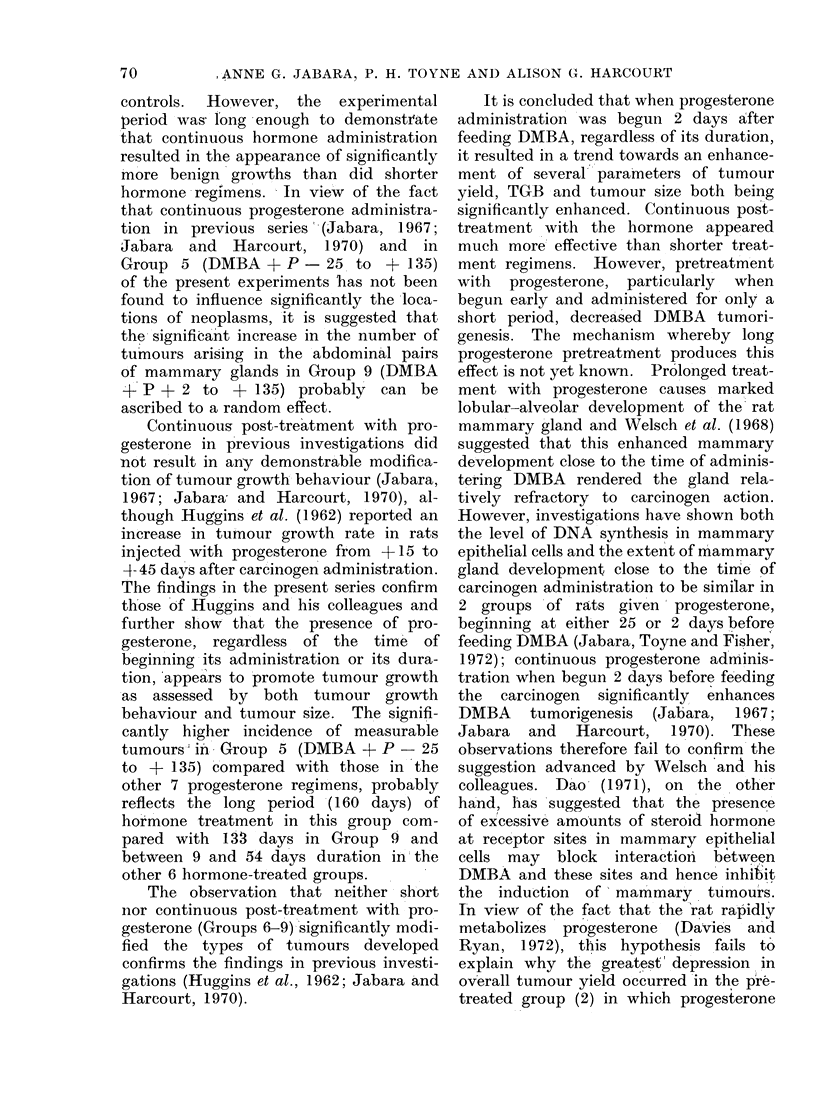

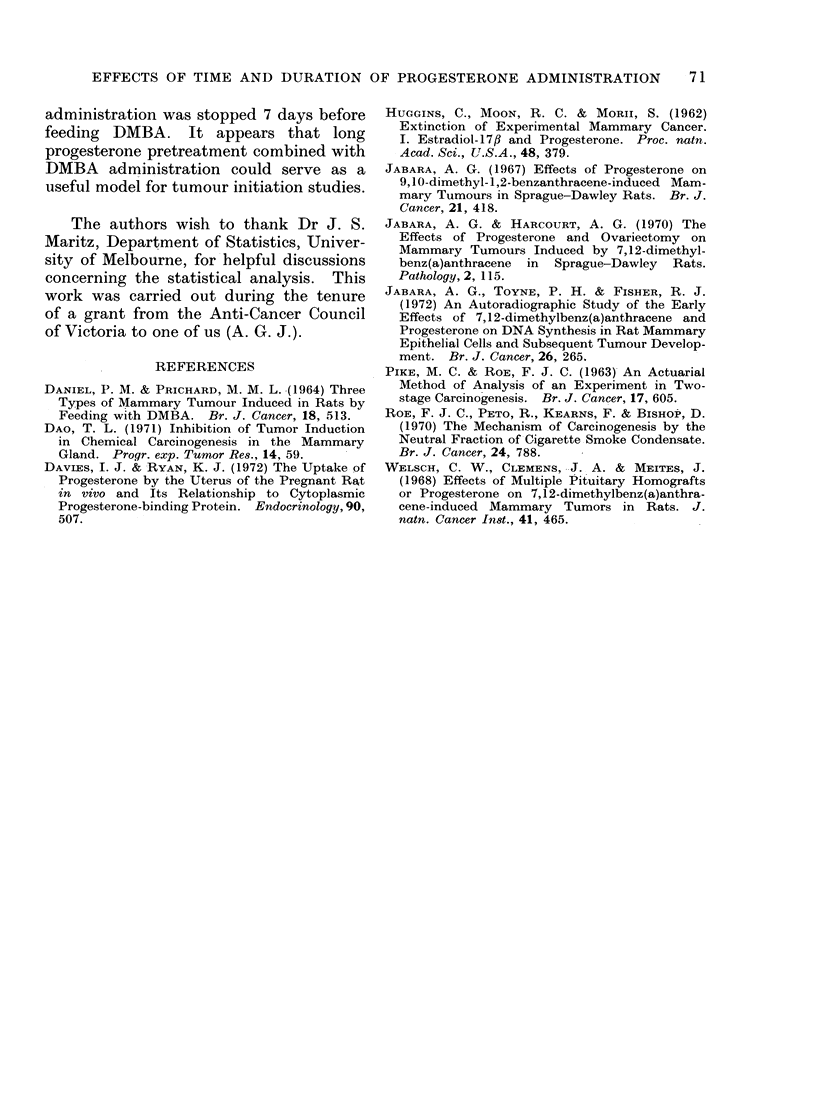


## References

[OCR_01122] DANIEL P. M., PRICHARD M. M. (1964). THREE TYPES OF MAMMARY TUMOUR INDUCED IN RATS BY FEEDING WITH DMBA.. Br J Cancer.

[OCR_01127] Dao T. L. (1971). Inhibition of tumor induction in chemical carcinogenesis in the mammary gland.. Prog Exp Tumor Res.

[OCR_01132] Davies I. J., Ryan K. J. (1972). The uptake of progesterone by the uterus of the pregnant rat in vivo and its relationship to cytoplasmic progesterone-binding protein.. Endocrinology.

